# Novel mutations of *STXBP2* and *LYST* associated with adult haemophagocytic lymphohistiocytosis with Epstein-Barr virus infection: a case report

**DOI:** 10.1186/s12881-019-0765-3

**Published:** 2019-02-19

**Authors:** Lingshuang Sheng, Wei Zhang, Jia Gu, Kefeng Shen, Hui Luo, Yang Yang

**Affiliations:** Department of Hematology, Tongji Hospital, Tongji Medical College, Huazhong University of Science and Technology, Wuhan, 430030 Hubei China

**Keywords:** Haemophagocytic lymphohistiocytosis, Digenic mutation, *STXBP2*, *LYST*, Chronic active Epstein-Barr virus infection, Case report

## Abstract

**Background:**

Haemophagocytic lymphohistiocytosis is a life-threatening disease resulting from primary or secondary hyper-inflammatory disorders. The typical symptoms include persistent fever, splenomegaly, cytopenia and significant elevation of serum ferritin.

**Case presentation:**

We report a 30-year-old Chinese female patient who was diagnosed with chronic active Epstein-Barr virus infection more than 9 months prior and has since been presenting with cutaneous lymphoproliferative disorders mimicking hydroa vacciniforme and subsequent haemophagocytic lymphohistiocytosis. Exome sequencing suggested novel digenic heterozygous *STXBP2* (c.592A > C, p.Thr198Pro) and *LYST* (c.830A > T, p.His277Leu) mutations.

**Conclusions:**

This is the first case report in which adult HLH was associated with novel digenic mutations of *STXBP2* and *LYST* combined with Epstein-Barr virus infection. It could also be the first polygenic model report, given that the pathogenicity of other mutated genes still remains unclear. We additionally conducted an in-depth, two-generation pedigree analysis to further illustrate the mode of inheritance in this case.

## Background

Haemophagocytic lymphohistiocytosis (HLH) is a fatal disease resulting from primary or secondary hyper-inflammatory disorders with symptoms of persistent fever, splenomegaly, cytopenia and significant elevation of serum ferritin. Primary HLH, also known as familial HLH (FHL), is caused by a deficiency of specific genes, namely, *perforin1* (*PRF1*), *UNC13D*, *syntaxin 11* (*STX11*), *syntaxin binding protein 2* (*STXBP2*), *lysosomal trafficking regulator* (*LYST*), *RAB27A*, *AP3B1*, *SH2D1A* and *X-linked inhibitor of apoptosis* (*XIAP*) as well as some other unknown genes [1–5]. Secondary HLH is a type of acquired disease associated with infections, malignancies or autoimmune disorders. *STXBP2*, which is regarded as being associated with HLH type 5 (FHL-5), is located on chromosomal arm 19p13 and encodes the *Munc18–2* protein that takes part in vesicle docking and fusion [6]. On the other hand, *LYST* (1q42) mutation is the cause of *Chediak-Higashi Syndrome* (CHS) and is involved in vesicle trafficking [7]. In fact, although FHL follows autosomal recessive inheritance, a heterozygous mutation may also lead to late-onset HLH in elderly patients according to the historical reports and our clinical experience [8–10]. Digenic and polygenic mutation models may demonstrate synergistic defects in cytotoxic pathways to offset the relatively low pathogenicity of heterozygotes and could lead to clinical HLH [11, 12].

Hereby, we report a Chinese female patient diagnosed with chronic active Epstein-Barr virus infection (CAEBV) more than 9 months earlier; the patient presented with cutaneous lymphoproliferative disorders mimicking hydroa vacciniforme and subsequent HLH. Exome sequencing results suggests novel digenic heterozygous *STXBP2* (c.592A > C) and *LYST* (c.830A > T) mutations.

## Case presentation

**Fig. 1:**
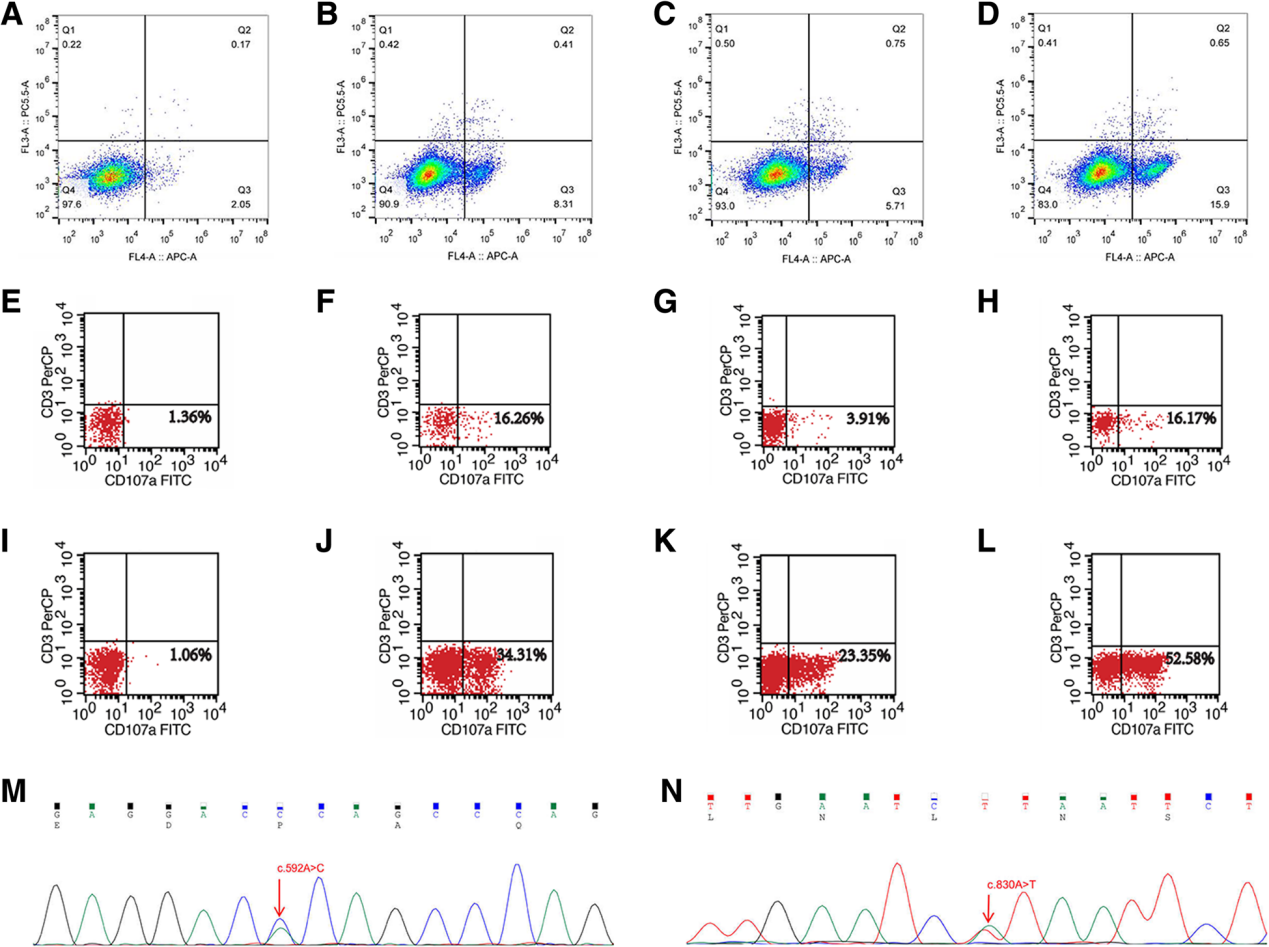
Target cell (K562-EGFP) apoptosis indicating NK cell killing activity examined using flow cytometry (Annexin V-APC, propidium iodide-PC5.5): **a** Natural apoptosis background of target cell. **b** Target cell apoptosis of the patient. **c** Target cell apoptosis of her mother. **d** Target cell apoptosis of her father. CD107a expression level indicating NK cell degranulation examined using flow cytometry (CD107a-FITC, CD3-PerCP): Resting **e** and activated (**i**) CD107a level of control group. Resting (**f**) and activated (**j**) CD107a level of the patient. Resting (**g**) and activated (**k**) CD107a level of her mother. Resting (**h**) and activated (**l**) CD107a level of her father. Heterozygous mutations of STXBP2 (c.592A > C, p.Thr198Pro) (**m**) and LYST (c.830A > T, p.His277Leu) (**n**) detected in DNA sequence of the patient’s mother by Sanger sequencing

The 30-year-old Han Chinese female patient was admitted to our hospital due to symptoms of fatigue and recurrent high-grade fever (> 39 °C) with a 4-month duration. She had presented with cutaneous lymphoproliferative disorders mimicking hydroa vacciniforme since the age of three and was diagnosed with CAEBV at Nanjing Drum Tower Hospital more than 9 months earlier. She experienced a spontaneous abortion 4 months ago. One month before her hospital visit, the patient underwent splenectomy at Nanjing for uncontrolled splenomegaly, and her postoperative pathology diagnosis suggested hypersplenism and EBV infection. She was noted to have oedematous swelling of the cheeks, eyelids and lips, and coexistent skin lesions, liver damage, pancytopenia with white blood cell (WBC) count of 1.90 × 10^9^/L, hypofibrinogenemia, plasma EBV-DNA 3.26 × 10^3^copies/L, EBV-DNA in peripheral blood mononuclear cells (PBMCs) of 5.93 × 10^4^ copies/L, ferritin 1090.7 μg/L, interleukin-6 (IL-6) level of 74.45 pg/mL and soluble interleukin-2 receptor (sIL-2R) level of 2083 U/mL. Her bone marrow examinations failed to identify any abnormal lymphocytes or haemophagocytosis. Peripheral blood cell sorting and EBV-DNA PCR suggested predominant EBV infection with 4.68 × 10^5^ copies per 2 × 10^5^ T lymphocytes and 1.17 × 10^5^ copies per 2 × 10^5^ NK cells. NK cell killing activity decreased to 6.50% (normally ≥15.11%) (Fig. 1b), and the expression levels of activated CD107a (for assessing NK cell degranulation) decreased to 33.24% (normally ≥40%) (Fig. 1j). Exome sequencing demonstrated the presence of novel digenic heterozygous *STXBP2* (c.592A > C) and *LYST* (c.830A > T) mutations as well as some variants of unknown significance with HLH (Table 1, Fig. 1). Two-generation pedigree analysis using Sanger sequencing showed that the mutations were inherited from her parents, and NK cell function tests for her parents were conducted as well (Table 2, Fig. 1). We noticed that her mother had an NK cell dysfunction which was even more severe than that of the patient herself, while her father’s NK cell functions were all normal. It still remains unclear why the patient’s mother did not experience any clinical symptoms all the way through, and we formulated our assumption in Discussion and Conclusions section. Because seven of the eight criteria of HLH-2004 were met [13], the patient was finally identified to have secondary HLH. X-linked lymphoproliferative disease (XLP) is a secondary disease caused by immunodeficiency-mediated EBV infection. Individuals with XLP-1 are uniquely sensitive to diseases caused by EBV, which otherwise runs a fairly benign course in most healthy individuals. HLH represents 60% of all the disease clinical features while the age of onset is within the range of 0.5–40 years old [14]. The symptoms of HLH secondary to XLP is very similar to our case. However, the patient in our case cannot be diagnosed with XLP since we found that she and her parents had no SH2DIA or XLP1 mutations via WES and Sanger sequencing tests.

**Table 1:** Detected variations in the patient with HLH

Gene	Position in reference genome hg19/GRCh37	Nucleotide and amino acid change	Zygosity (variant allele frequency)	ID in dbSNP (population based allele frequency^a^)	Associated features^b^
*STXBP2*	19:7706933 NM_006949	c.592A > C p.Thr198Pro	Heterozygous (0.542)	rs760187284 (2/18368, 0.0001089)	Fever, Hepato-Splenomegaly (HSMG), HLH, Cytopenias
*LYST*	1:235973288 NM_001301365	c.830A > T p.His277Leu	Heterozygous (0.493)	rs1247313496 (2/18288, 0.0001094)	Partial albinism, recurrent infections, fever, HSMG, HLH Giant lysosomes, neutropenia, cytopenias, bleeding tendency, progressive neurological dysfunction
*LRBA*	4:151827481 NM_006726	c.1570G > A p.Gly524Ser	Heterozygous (0.447)	rs776254567 (57/19930, 0.002860)	Recurrent infections, inflammatory bowel disease, autoimmunity; EBV infections
*AIRE*	21:45708288 NM_00383	c.599C > T p.Pro200Leu	Heterozygous (0.496)	rs140196414 (9/19942, 0.0004513)	Autoimmunity: hypoparathyroidism hypothyroidism, adrenal insufficiency, diabetes, gonadal dysfunction and other endocrine abnormalities, chronic mucocutaneous candidiasis, dental enamel hypoplasia, alopecia areata Enteropathy, pernicious anemia
*IRF8*	16:85942708 NM_002163	c.287C > T p.Thr96Met	Heterozygous (0.457)	rs145048966 (0/19954, 0.000)	Susceptibility to Mycobacteria

^a^ “population based allele frequency” is east Asian population based allele frequency based on gnomAD (http://gnomad-old.broadinstitute.org)

^b^ “Associated features” is based on the classification of primary immunodeficiencies compiled by the Primary Immunodeficiency Expert Committee (PID EC) of the International Union of Immunological Societies (IUIS) [22, 23]

**Table 2:** Two-generation analysis of mutation genes and NK cell function

		Patient	Mother	Father
Mutation genes	*STXBP2*	+	+	–
	*LYST*	+	+	–
	*LRBA*	+	+	–
	*AIRE*	+	–	+
	*IRF8*	+	–	+
NK cell function	Killing activity (normally ≥15.11%)	6.50%	4.97%	16.18%
	Activated CD107a level (normally ≥40%)	33.24%	22.60%	51.20%
	Resting CD107a level (normally ≥5%)	14.90%	3.14%	15.87%

+, mutant; −, wild type

Following diagnosis, the patient was treated with persistent small dose of dexamethasone (0.1 mg/kg·d^− 1^). We were able to manage the disease in the beginning, with a decreased ferritin level of 5951 μg/L, IL-6 level of 17.78 pg/mL and normal body temperature. However, her condition was out of control one month later with the appearance of recurrent high-grade fever, rising levels of inflammatory factors, macrophage activation syndrome (MAS) and capillary leak syndrome (CLS), and we switched her therapy to the HLH-2004 protocol with etoposide and dexamethasone. DEP regimens (liposomal doxorubicin, etoposide and methylprednisolone) were also provided when HLH relapsed for the second time [15]. Ferritin levels peaked at 3222.2 μg/L and subsequently began to decline. Currently, the HLH of this patient has been well-controlled for a month and she is currently waiting for haematopoietic stem cell transplantation (HSCT).

For Whole Exome Sequencing (WES), DNA samples were isolated from peripheral blood. The genomic library of the proband was recovered for exome enrichment with Agilent Sure Select Human Exon v7 and was sequenced by Illumina HiSeq2500 with an average 300x coverage. The Broad Institute’s Genome Analysis Toolkit was applied during the data analysis. Reads were aligned with the Illumina Chastity Filter and the Burrows Wheeler Aligner. Variants were identified by the GATK UnifiedGenotyper module. For quality control measures, coverage per base was 351x, and Q30 percentage was 92.71%. For filtering strategy: First, generated variants were locally annotated with Annovar software under Linux system. Next, only mutations affecting amino acid sequence were filtered for further analysis (including missense, nonsense, frameshift/non-frameshift insertion/deletion, splice-site mutation and other complex mutations). Afterwards, variants with frequency in Han population no less than 0.01 were filtered out. Then variants failed to pass quality control (reads < 20, quality< 30, or variants with significant strand bias) were filtered out. The mutated genes were filtered out based on classification of primary immunodeficiencies compiled by the Primary Immunodeficiency Expert Committee (PID EC) of the International Union of Immunological Societies (IUIS) [16, 17], and further tested by Sanger sequencing. Splice site variants were taken into consideration in this filter strategy while promoter regions were not. WES study was not performed on the proband’s parents. Only potential pathogenic mutations in Primary Immunodeficiency (PID) associated genes (Table 1) of the proband were identified in the proband’s parents using Sanger sequencing.

## Discussion and conclusions

This is the first case report of adult HLH associated with novel digenic mutations of *STXBP2* and *LYST*, in combination with EBV infection. It is possibly the first polygenic model report as well, since the pathogenicity of other mutated genes are not yet clear. We also conducted an in-depth two-generation pedigree study to clarify the mode of inheritance in this case.

Exome sequencing results suggested that the patient has *LPS-Responsive Beige-Like Anchor Protein* (*LRBA*), *Autoimmune Regulator Gene* (*AIRE*) and *Interferon Regulatory Factor 8* (*IRF8*) mutations in addition to *STXBP2* and *LYST* mutations. Inherited from her mother, the *LRBA* mutation could lead to autoimmune diseases and EBV lymphoproliferative disease as reported in another case [18]. All the other mutations of the patients were inherited from her father. *AIRE* mutation is associated with autoimmunity diseases [19], while *IRF8* mutation is associated with dendritic cells (DC) maturation and influences NK cell functionalities [20]. Taken together, it is difficult to verify whether or not these mutated genes are all involved in this patient’s EBV infection and HLH. Whether this case follows a digenic or a polygenic model also remains a question. However, her father and uncle (with *IRF8* mutation detected) are not eligible to be transplantation donor as their mutated genes could also be associated with HLH occurrence. Unrelated donor allogeneic haematopoietic stem cell transplantation (URD-HSCT) should be taken into consideration for every patient, and donors have to receive NK cell functionality testing as well in order to confirm that the procedure could be effective thorough elimination of deficient NK cells in the patient body and thus prevent relapse [14]. Actually, among the cases (EBV+ T/NK-cell lymphoproliferative disease, infectious mononucleosis, EBV+ B-cell lymphoma, Hodgkin lymphoma, posttransplant lymphoproliferative disorders and EBV+ T/NK-cell lymphoma) we collected, the NK cell activity of most patients combined with EBV related diseases were suppressed (61/88, 69.3%), only few of them were normal. Moreover, every HLH patient (22)'s NK cell functionality was suppressed or even absent, which slowly recovered once HLH symptoms were controlled (Author, unpublished data, 2018).

As for the reason why the proband’s mother did not develop any clinical sign, here’s our assumption: Firstly, the patient’s mother is heterozygote. Differing from pediatric HLH that are typically due to homozygous mutations, the vast majority of EBV related adult HLH attacks are caused by heterozygous mutations. Secondly, the patient inherited other potential pathogenic genes from her father as well, except for the 2 genes inherited from her mother that are most likely pathogenic. Last, adult HLH is a type of disease which is generally caused by genetic mutations along with other acquired factors, for instance infections, tumors, etc. The patient in this case had had an abortion before being caught in the fever, which might be a precipitating factor of the HLH occurrence. We tested her mother’s plasma EBV-DNA and the result was negative (1.35 × 10^2^/L in peripheral blood mononuclear cell). The low-copy EBV of her mother appears to be a solid evidence why she did not progress into HLH or any relevant disease phenotypes, despite being found with STXBP2, LYST and other heterozygous mutations along with declined NK activity, but it could be deduced that the mother falls under the high-risk population of secondary HLH. EBV infection is the most frequent factor that triggers subsequent HLH [21]. For people with diagnosed immune deficiencies which increase their EBV susceptibility, it would be particularly difficult to achieve full virus elimination. As a result, the persistence of EBV infection would contribute to HLH and NK/T lymphoma occurrence [14]. In this case, unknown mechanisms that are able to inhibit EBV activity were probably involved in her mother’s case, and the persistent EBV infection of the patient was possibly the reason why she came to a completely distinct consequence as compared with her mother.

Although progress has been made in understanding HLH, it is still a life-threatening disease today. We report this case for the illustration of possible digenic and polygenic mode of inheritance of HLH that have been previously confirmed in the mouse model only [12]. We are looking forward to advancing our understanding and offering a higher level of individualization of HLH treatments in the future, apart from classic chemotherapy and HSCT. Cord blood NK cell transplantation is expected to be capable of repairing damaged NK cells [22, 23], and monoclonal antibodies (mAb), for example the anti-sCD25 mAb, could potentially target certain cytokines that are perceived as unmanageable [24]. In the future, customized gene therapy may provide solutions for the treatment of HLH.

## Abbreviations

*AIRE*: Autoimmune regulator gene
CAEBV: Chronic active Epstein-Barr virus infection
CHS: Chediak-Higashi Syndrome
CLS: Capillary leak syndrome
DC: Dendritic cell
FHL: Familial haemophagocytic lymphohistiocytosis
HLH: Haemophagocytic lymphohistiocytosis
HSCT: Haematopoietic stem cell transplantation
IL-6: Interleukin-6
*IRF8*: Interferon regulatory factor 8
*LRBA*: Lipopolysaccharide-responsive beige-like anchor protein
*LYST*: Lysosomal trafficking regulator
mAb: Monoclonal antibodies
MAS: Macrophage activation syndrome
PBMC: Peripheral blood mononuclear cell
PID EC: Primary Immunodeficiency Expert Committee
PID: Primary Immunodeficiency
*PRF1*: Perforin 1
sIL-2R: Soluble interleukin-2 receptor
*STX11*: Syntaxin 11
*STXBP2*: Syntaxin binding protein 2
URD-HSCT: Unrelated donor allogeneic haematopoietic stem cell transplantation
WBC: White blood cell
WES: Whole Exome Sequencing
*XIAP*: X-linked inhibitor of apoptosis
XLP: X-linked lymphoproliferative disease
